# Of Skiers, Smokers And Amelioration Of The World

**DOI:** 10.1186/1617-9625-3-1-3

**Published:** 2005-12-15

**Authors:** David Bernhard

**Affiliations:** 1 Division Experimental Pathophysiology and Immunology, Biocenter – Innsbruck Medical University, Austria

## 

Dear Colleagues,

From the "passive-sports" point of view, football (soccer) is the number 1 sport in Austria. Unfortunately, the Austrian national football team is anything but well known for playing football, and honestly, from time to time I thought about starting a public opinion poll to abolish the national Austrian football team. However, we (the Austrians) are doing our best to compensate for this annoyance, and in fact we found things that we are really good in: SKIING. Yes, the Austrians are definitely the number one in the world. – at least for the moment....

Only recently, it seems that we have also managed to become European champions with ambitions to truly become world champions in another thing: SMOKING. According to the World Health Organization (WHO), Austria has reached **47% smokers in the adult **population and a horrific **31.5% smokers among youngsters **http://data.euro.who.int/?TabID=2404. Years and years of hard work doing nothing but leaving the field to the tobacco industry, finally enabled the Austrian politicians and the population to achieve this goal. In summary, Austria is probably the worst example among the developed countries concerning anti-smoking campaigning, protection of non-smokers from passive smoking, and preventing people from starting smoking as well as helping people to quit smoking.

In vast contrast, a number of countries like the USA and Sweden have made excellent progress in protecting non-smokers, and in reducing the smoking prevalence in their population below 20%. However, as you all know the situation in the world and especially among the poor and the young is becoming worse. Still, in the view of many scientists smoking is "the most easily preventable risk factor" for diseases like cancer, chronic obstructive pulmonary diseases, and cardiovascular diseases. Although this may be true from a theoretical point of view, the statistics of the World Health Organisation tell a different story, estimating that by 2020 smoking will become the largest single health problem in the world, causing 8.4 million deaths a year. Why should reducing the number of smokers be easier compared to reducing the number of people suffering from obesity? Of course there is no sense in charging up one health problem against another, but we have to face the truth that smoking is not "easily preventable". Without doubt, a lot of preventive strategies and quitting programs are successful and smoking prevention should and will clearly stay the number one in the fight against tobacco addiction and diseases. I am convinced that in the end, the only tool that may completely solve the problem in the future is prevention, but facing up to the WHO statistics for a moment, we have to think about additional strategies. E.g.: In the past, the carcinogenic potential of cigarette smoke and smoke chemicals has been analysed in great detail – this knowledge could now be used to generate a "less harmful cigarette". Of course we do not need "less harmful cigarettes" in the public mind but we should think about extending the research efforts on reducing the deleterious health effects of cigarette smoke, to counteract the expected increase in the incidence of smoking caused diseases. Having the goal of a tobacco free society in our minds, we must not forget that humans are suffering from tobacco caused diseases now, tomorrow and also in the next decades, which can in part be prevented by reducing the toxicity of cigarette smoke.

I am aware that this is a very controversial issue, and that only recently the tobacco industry has claimed to bring "healthier" cigarette to the market, which is without doubt a primitive tobacco commercial. Although, I am not completely sure if it would be advantageous to have a real "less harmful cigarette" (loss of determent), I am sure that leaving the toxicity of cigarettes as it is will cause a lot of morbidity and mortality.

With this article, I would like to start a discussion on the following issue: Why shouldn't it make sense to intensify the efforts to reduce the number of active and passive smokers AND reduce the toxicity of cigarette smoke?

## Competing interests

The authors declare that they have no competing interests.

